# Phase-Dependent Photocatalytic Activity of Nb_2_O_5_ Nanomaterials for Rhodamine B Degradation: The Role of Surface Chemistry and Crystal Structure

**DOI:** 10.3390/nano15110846

**Published:** 2025-06-01

**Authors:** Aarón Calvo-Villoslada, Inmaculada Álvarez-Serrano, María Luisa López, Paloma Fernández, Belén Sotillo

**Affiliations:** 1 Department of Material Physics, Faculty of Physics, Complutense University of Madrid, 28040 Madrid, Spain; aaroncal@ucm.es (A.C.-V.); arana@ucm.es (P.F.); 2 Department of Inorganic Chemistry, Faculty of Chemistry, Complutense University of Madrid, 28040 Madrid, Spain; ias@ucm.es (I.Á.-S.); marisal@ucm.es (M.L.L.)

**Keywords:** niobium oxide, X-ray diffraction, Raman spectroscopy, photocatalysis

## Abstract

Niobium oxides are promising materials for catalytic applications due to their unique structural versatility and surface chemistry. Nb_2_O_5_ nanomaterials were synthesized via a solvothermal method at 150 °C using niobium oxalate as a precursor. A comprehensive characterization of the material was performed using electron microscopy, X-ray diffraction, and Raman spectroscopy. The as-prepared nanoparticles primarily crystallized in a mixture of the TT-Nb_2_O_5_ phase (TT from the German Tief-Tief, meaning “low-low”) and niobic acid, while subsequent thermal treatment at 900 and 1100 °C induced a phase transformation to T-Nb_2_O_5_ and H-Nb_2_O_5_, respectively (T from the German Tief, meaning “low”, and H from Hoch, meaning “high”). The as-prepared samples consist of micro-coils composed of interconnected nanometer-scale fibers, whereas the morphology changes into rods when they are treated at 1100 °C. The photocatalytic performance of the nanoparticles was evaluated by comparing the as-prepared and thermally treated samples. The as-prepared nanoparticles exhibited the highest photocatalytic activity under visible illumination, achieving 100% degradation after 180 min. More interestingly, the treatment of the as-prepared material with H_2_O_2_ modified the surface species formed on the Nb_2_O_5_, altering the photocatalytic behavior under various illumination conditions. This sample showed the highest photocatalytic activity under UV illumination, reaching 100% degradation after 75 min. On the other hand, the calcined samples are practically inactive, attributed to the loss of active catalytic sites during thermal treatment and phase transformation.

## 1. Introduction

Niobium is a transition and refractory metal that shows superior hardness, durability and resistance to corrosion. These outstanding properties have defined the real-life applications in which it is used: high-tech superalloys, protective coatings and capacitors. Another important field where it shows outstanding performance is protective coatings for medical applications [1]. This is due to the formation of a protective oxide layer that increases the wear and corrosion resistance with high biocompatibility, low ion release, non-toxicity to the surrounding cells and non-inhibiting cell growth [2,3]. These outstanding properties of the niobium oxide compounds can also be employed for other applications; for example, in electrochemical devices to increase their lifetime [4] or in photocatalysis processes to clean pollutants without producing any biological hazard [5].

Niobium oxides have a huge diversity of compositions and crystal structures [6], which hinders the development of a complete description relating the phases of these oxides with their physico-chemical properties. In particular, niobium pentoxide (Nb_2_O_5_) undergoes a sequence of phase transformations depending on the temperature during thermal treatment, starting from its hydrated precursor, the so-called niobic acid (Nb_2_O_5_·nH_2_O). Nb_2_O_5_·nH_2_O typically exhibits an amorphous structure, composed of hydrated Nb-O polyhedra with short-range order [7]. When niobic acid is subjected to mild heating around 300–500 °C, Nb_2_O_5_ crystallizes into the so-called TT-Nb_2_O_5_ (pseudo-hexagonal phase), characterized by a lower degree of structural order. Further heating to around 600–900 °C drives the transformation into T-Nb_2_O_5_, a more ordered and orthorhombic structure. The temperature range at which the phase transformation is produced is also determined by the precursor used [6]. Finally, at even higher temperatures, typically above 900–1000 °C, T-Nb_2_O_5_ converts into the highly stable H-Nb_2_O_5_, which is monoclinic and represents the thermodynamically favored form of Nb_2_O_5_. Each phase transition is marked by gradual improvements in crystallinity, significantly influencing the material’s properties. The nomenclature of each polymorph used in this study originates from the first description provided by Brauer and consolidated by Schäfer et al. [8,9], where the TT-, T-, and H- prefixes correspond to the low-low (Tief-Tief), low (Tief), and high-temperature (Hoch) phases of Nb_2_O_5_, respectively.

In Nb_2_O_5_-based materials, Lewis acid sites (LAS) are typically associated with unsaturated niobium sites or surface oxygen vacancies, whereas Brønsted acid sites (BAS) arise from surface hydroxyl groups capable of donating protons [10]. Additionally, highly distorted NbO_6_ octahedra can lead to Nb=O bonds and oxygen deficiencies, exposing Nb ions that function as LAS [11,12]. In contrast, Nb–O bonds are more closely related to BAS. Niobic acid (Nb_2_O_5_·nH_2_O), an amorphous precursor of Nb_2_O_5_, predominantly contains distorted [NbO_6_] octahedra linked to BAS and [NbO_4_] tetrahedra that act as LAS through the formation of NbO_4_–H_2_O adducts. The pseudohexagonal TT-Nb_2_O_5_ phase also features highly distorted NbO_6_ octahedra, contributing to the presence of LAS. As the crystal phase evolves from a lower degree of structural order towards a higher degree of order, the surface properties of the material are altered. Lower-temperature phases, like niobic acid or TT-Nb_2_O_5_, usually have higher surface areas and more structural defects [12], providing abundant active sites for catalytic reactions, especially those requiring acid sites or redox activity. Thus, with increasing synthesis temperatures (e.g., transitioning from niobic acid to monoclinic H-Nb_2_O_5_), the concentration of surface OH groups and active BAS decreases. While factors like pH and precursor choice also influence the final material properties, it is generally accepted that different polymorphs exhibit distinct catalytic behaviors [13]. Notably, niobic acid and TT-Nb_2_O_5_ have been shown to outperform orthorhombic (T-phase) and monoclinic (H-phase) Nb_2_O_5_ in photocatalytic applications [14]. It was reported that an increase in the temperature of thermal treatment reduces the photocatalytic efficiency of Nb_2_O_5_ for dye degradation [13,15], although no strong variation of the bandgap is reported upon phase transformation [16].

Hydrogen peroxide (H_2_O_2_) is known to have an impact on the catalytic properties of Nb_2_O_5_. H_2_O_2_ has been added during the synthesis process [17] as well used to treat the material after being obtained [18,19,20]. It has also been added directly into the photocatalysis process to degrade Rhodamine B [21,22]. In these studies, the improvement in the photocatalytic efficiency is related to the formation of reactive oxygen species (ROS) at the surface, mainly peroxo (O_2_^2−^) and hydroxyl (OH) groups [20].

In this work, we prepared Nb_2_O_5_ nanomaterials showing different crystal structures. The solvothermal method and precursor used allowed us to obtain micro-coils composed of interconnected nanometer-scale fibers, a morphology different from the nanoparticles or commercial material reported in previous works [15,17,18,19,20,21]. The photocatalytic performance of the nanoparticles was evaluated by comparing the as-prepared and thermally treated samples. We studied also the effect of treating the surface of the samples with hydrogen peroxide (H_2_O_2_). Finally, we analyzed the influence of using both UV and visible light in the photocatalytic process, to make use of the full solar spectrum [23].

## 2. Materials and Methods

Nb_2_O_5_ nanoparticles were prepared by a solvothermal method, niobium oxalate hydrate (V) (Nb(HC_2_O_4_)_5_·mH_2_O, Alfa Aesar, Ward Hill, MA, USA) as a precursor. Thermogravimetric analysis shows that the hydration of the precursor is negligible (m~0). 1 g of niobium oxalate was first stirred in 10 mL ethanol for 15 min at room temperature. Then, 13 mL of distilled water is added, and the solution is stirred for 30 min at room temperature till it looks completely transparent. The solution was poured into an autoclave and heated for 17 h at 150 °C. The white powder obtained was filtered and dried at 50 °C in a stove. Considering the mechanisms involved in the thermal decomposition of oxalate-based compounds, as reviewed by Dollimore [24] the decomposition of the precursor may be proposed as follows:

(1) $$2Nb{\left(HC_{2}O_{4}\right)}_{5}\;⟶Nb_{2}O_{5}\cdot nH_{2}O+10CO_{2}+{(5-n)H}_{2}O+10CO$$

It has been previously reported that the dehydration of niobic acid ($Nb_{2}O_{5}\cdot nH_{2}O$) occurs between 50 °C and 300 °C [25]. In our case, the additional water content may also originate from the aqueous environment within the autoclave. Therefore, the obtained sample, referred to as Nb_2_O_5_-as-prepared, is likely composed of a mixture of niobium oxides exhibiting varying degrees of hydration. Samples with high-temperature phases were obtained by heating the as-prepared Nb_2_O_5_ at 900 °C and 1100 °C in an oven. Additionally, as prepared sample was subjected to a treatment in H_2_O_2_ (solution 30% *w*/*w*, EssentQ). The white powders were immersed in H_2_O_2_ in a glass beaker, quickly changing to a light yellow color. Next, the beaker is heated until H_2_O_2_ is completely evaporated, and the yellowish powder is recovered. Four samples were used for our experiments, summarized in Table 1.

Different techniques were employed to perform the characterization of the samples obtained. Morphological characterization was performed using Scanning Electron Microscopy (SEM) in a FEI Inspect microscope (FEI, Hillsboro, OR, USA) working at 15 kV. Structural characterization of the samples was performed by means of X-ray Diffraction measurements (XRD), in a Bruker D8 Advance A25 diffractometer (Bruker, Billerica, MA, USA) or in a PANanalytical X’PERT POWDER diffractometer (Malvern Panalytical, Worcestershire, UK), in both cases using Cu-Kα radiation and with a step-in 2θ of 0.05°. Additionally, Raman spectroscopy was performed at room temperature in a Horiba Jobin Yvon LabRAM HR800 confocal microscope (Horiba, Kyoto, Japan), using the 632.8 nm line of a He-Ne laser (power of 17 mW at laser output). The laser was focused onto the sample with a 100× Olympus objective (0.9 NA), and the scattered light was collected using the same objective (backscattering configuration). The grating used to analyze the signal had 600 L/mm, and the signal was recorded with an air-cooled charge-coupled device camera (CCD). Exposure time ranges from 1 to 10 s depending on the sample.

Thermogravimetric analyses (TGA) were carried out under nitrogen by a Pyris thermogravimeter (PerkinElmer, Waltham, MA, USA). The analyses were performed under nitrogen at a heating/cooling rate of 10 °C min^−1^ in the 40–900 °C temperature range. High-resolution transmission electron microscopy (HRTEM) was performed in a JEOL 300FEG (JEOL Ltd., Tokyo, Japan). The composition of the obtained materials was established by semi-quantitative chemical analysis using energy-dispersive X-ray spectroscopy (EDX). The samples were prepared by crushing the powders under n-butanol and dispersing them over copper grids covered with a porous carbon film. Fourier Transform Infrared Spectra (FTIR) were recorded using a Perkin-Elmer UATR Two spectrophotometer in the range of 400–4000 cm^−1^ with a diamond accessory of Attenuated Total Reflection (ATR) mode. Diffuse reflectance spectroscopy (DRS) was performed in a UV-vis Jasco V770 spectrometer (Jasco Inc., Tokyo, Japan).

Finally, photocatalysis tests were performed using Rhodamine B as a pollutant simulator. The solution contains 2.5 ppm of the dye (Rhodamine B (RhB), Sigma-Aldrich (St. Louis, MO, USA), with a purity greater than or equal to 95%) in distilled water. The RhB concentration is selected to maintain a linear relationship between absorbance and concentration (in accordance with Beer’s Law) and to ensure enough RhB for studying photocatalytic kinetics. We checked the degradation of the dye by measuring absorption curves with the UV-vis Jasco V770 as a function of illumination time. The degradation experiments were performed with a catalyst load of 0.4 g/L, and aliquots of 5 mL of solution were collected every 15 min for the first 2 h and every 30 min thereafter. Prior to the illumination of the samples with the catalyst, the solution (Rhodamine B + catalyst) was maintained in the dark for 30 min to reach the adsorption equilibrium. The illumination was performed using commercial LED strip lights (2 strips of 25 W for UV (peak wavelength at 405 and 365 nm) and 2 strips of 35 W (Color Rendering Index (CRI) > 90) for simulating sunlight) purchased from growthejungle.com. They employ Samsung 6v and Osram 6v LED (Osram, Munich, Germany) in visible strips, and Seoul Viosys LED (Seoul Viosys Co., Seoul, Republic of Korea) for the UV strips. The emission spectra of the lamps are shown in Figure S1.

## 3. Results

**Figure 1:**
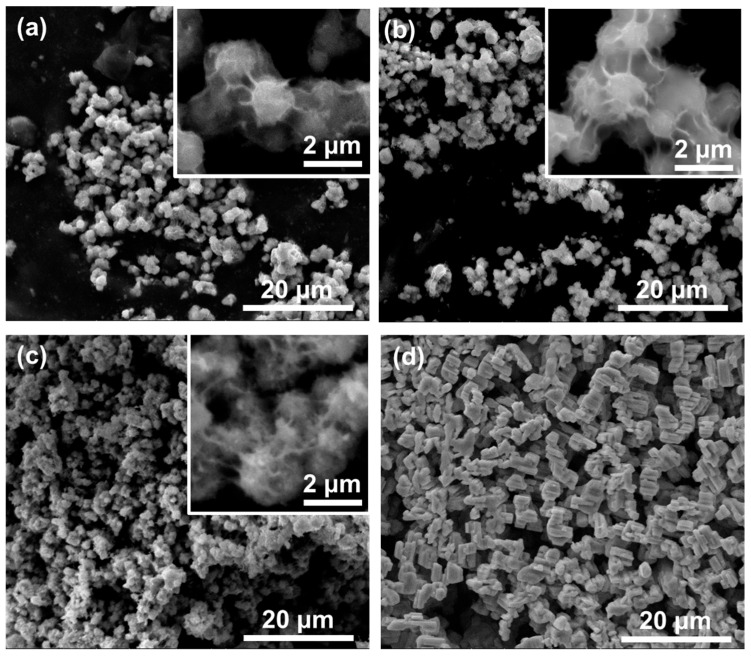
SEM images of the samples: (**a**) Nb_2_O_5_-as-prepared; (**b**) Nb_2_O_5_-H_2_O_2_; (**c**) Nb_2_O_5_-900 and (**d**) Nb_2_O_5_-1100.

SEM images of the four samples employed in this study are presented in Figure 1. Nb_2_O_5_-as-prepared image (Figure 1a) indicates that the powders are composed of particles of sphere-like shape with micrometric diameters. A closer inspection (Figure 1a inset) shows that a high density of fibers, with diameters of tens of nanometers and lengths of microns, covers the surface of the spheres. The samples treated with H_2_O_2_ (Figure 1b) or at 900 °C (Figure 1c) do not show any morphological modifications compared to the as-prepared sample. The main morphology variations appear upon heating at 1100 °C (Figure 1d). The spheres turn into micrometric rod-like particles, structures characteristic of the high-temperature crystal phases [26].

**Figure 2:**
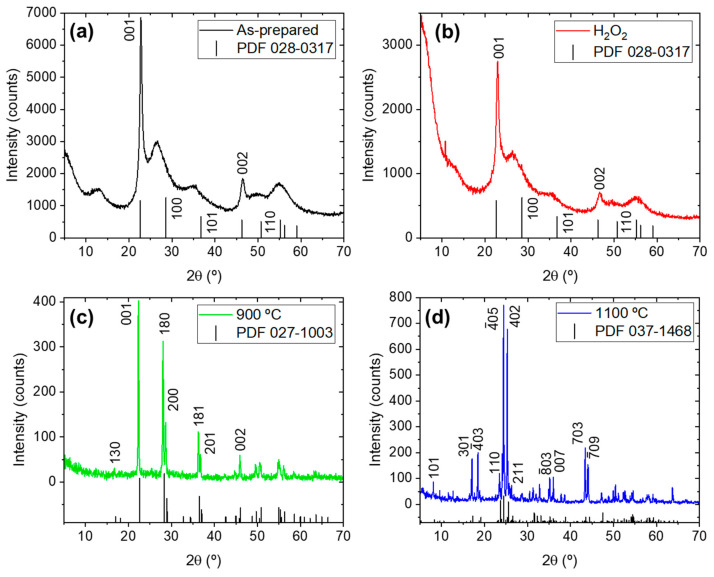
XRD measurements: (**a**) Nb_2_O_5_-as-prepared; (**b**) Nb_2_O_5_-H_2_O_2_; (**c**) Nb_2_O_5_-900; (**d**) Nb_2_O_5_-1100. The PDF files are indicated for each sample.

XRD measurements were made to determine the crystal phase of the four samples. The diffractograms are presented in Figure 2, and the phase transformation of Nb_2_O_5_ is clearly visualized upon heating. In the as-prepared sample (Figure 2a), reflections associated with TT-Nb_2_O_5_ are detected at 22.8°, 35.3°, 46.5°, 50.3° and 55.1° (PDF No. 028-0317). Additionally, the broad maxima located between 24–30° is related to niobic acid (Nb_2_O_5_·nH_2_O) [17]. Then, the as-prepared is composed of a combination of a TT phase and an amorphous phase, in alignment with the intended synthesis route. The prominence of some of the reflections aligns with the presence of a layered structure in the material [27]. The sample treated with H_2_O_2_ shows no evidence of crystal phase modification, apart from a reduction in the overall intensity and the increase in the relative intensity of the amorphous maxima (24–30°) relative to the first reflection (at 22.8°) of TT-Nb_2_O_5_ (Figure 2b). Upon heating at 900 °C, the reflection maxima are all ascribed to T-Nb_2_O_5_ (Figure 2c, PDF No. 027-1003), whereas the reflections of H-Nb_2_O_5_ are identified in the sample treated at 1100 °C (Figure 2d, PDF No. 037-1468). These transformations were expected according to the literature [6].

**Figure 3:**
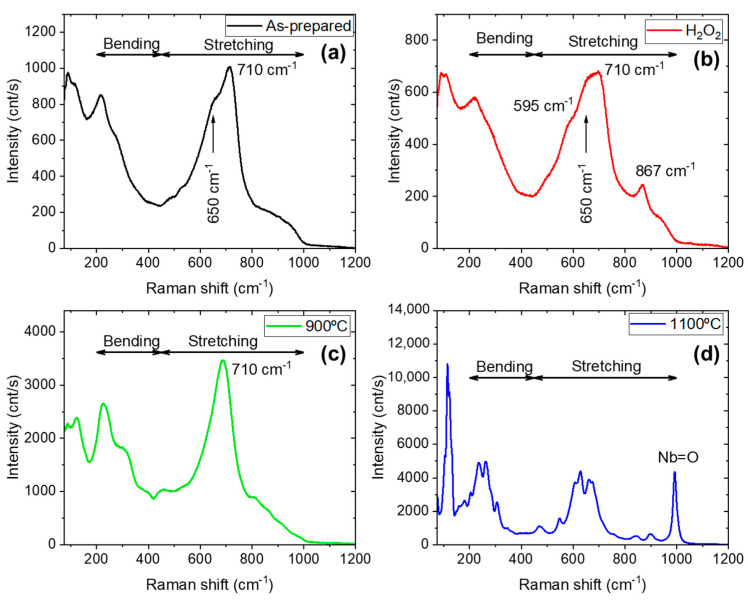
Raman spectra: (**a**) Nb_2_O_5_-as-prepared; (**b**) Nb_2_O_5_-H_2_O_2_; (**c**) Nb_2_O_5_-900; (**d**) Nb_2_O_5_-1100.

Additional information can be found about the crystal structure from the Raman spectra (Figure 3). The recorded spectra are different from the niobium oxalate precursor (Figure S2), hence confirming the transformation of the starting material. In agreement with the results of the XRD measurements, the Raman spectrum of the as-prepared sample shows characteristic features of T-Nb_2_O_5_ (Figure 3a). The band with maxima at 710 cm^−1^ can be ascribed to the stretching vibrations of [NbO_6_] octahedra. This position is expected for the TT phase [14]. The 215 cm^−1^ band is related to the bending modes of the octahedra. A broad shoulder between 600 and 1000 cm^−1^ is detected, related to the presence of the amorphous niobic acid in the sample [14], reflecting the distribution of [NbO_6_], [NbO_7_], and [NbO_8_] polyhedra in this phase.

Upon treatment with H_2_O_2_, the Raman spectrum shows new features (Figure 3b): a shoulder at 595 and a peak at 897 cm^−1^. Additionally, the bands exhibit increased width, which can be associated with the increased disorder in this sample [14], also observed in XRD. In fact, niobic acid shows the stretching vibration band at about 650 cm^−1^, consistent with the shoulder observed near the 710 cm^−1^ maxima, which is more pronounced in the spectrum of Figure 3b [14,16]. The peak at 867 cm^−1^ arises from the O-O vibration of the peroxo species (O_2_^2−^) formed at the surface of the material [19,20]. The shoulder at 595 cm^−1^ may arise from stretching modes of Nb coordinated with the peroxo ligands [20]. This observation confirms the formation of peroxo species on the surface of the material.

The Raman spectra of the samples calcinated at 900 °C and 1100 °C match the expected features for T-Nb_2_O_5_ (Figure 3c) and for H-Nb_2_O_5_ (Figure 3d) [14,28], as supported by XRD. As temperature increases, the stretching vibrations of the octahedra cover a narrower range of frequencies (Figure 3c) and sharper peaks appear as corresponding to the most ordered structure (Figure 3d). In all cases, modes related to the bending vibration (200–450 cm^−1^ range), and the stretching vibrations (450–900 cm^−1^ range) of the [NbO_6_] octahedra (with different degrees of distortion) are observed [14,29,30]. Moreover, for the 1100 °C sample, a peak at 992 cm^−1^ is ascribed to Nb=O vibrations. We tested also the effect of H_2_O_2_ treatment of samples calcinated at 900 and 1100 °C, but no modifications compared to the spectra presented in Figure 3c,d were observed.

**Figure 4:**
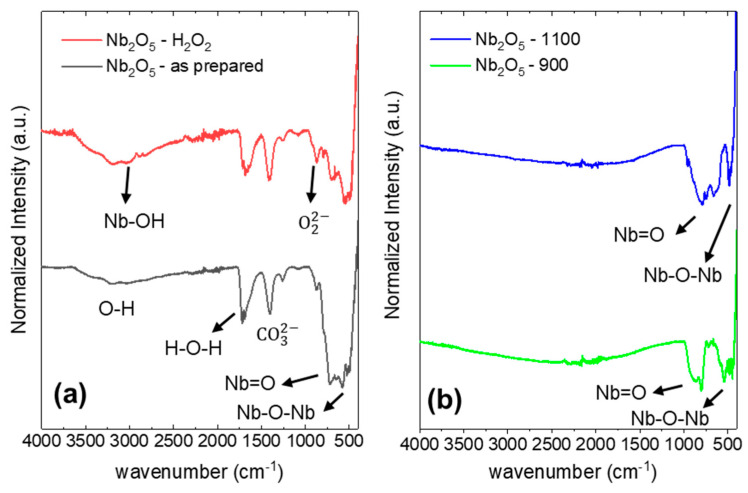
FTIR spectra of (**a**) Nb_2_O_5_-as-prepared and Nb_2_O_5_-H_2_O_2_, (**b**) Nb_2_O_5_-900 and Nb_2_O_5_-1100.

Complementary information can be extracted from the FTIR measurements of the different samples. Figure 4 shows the FTIR spectra corresponding to the as-prepared sample and after treatment with H_2_O_2_ (Figure 4a), together with the corresponding spectra of the sample after being treated up to 900 and up to 1100 °C (Figure 4b). The FTIR spectrum of the as-prepared sample exhibits characteristic bands commonly reported for niobium oxide materials synthesized via the solvothermal method [31,32]. The most prominent vibrational peak appears at 715 cm^−1^, corresponding to the stretching vibration of the Nb=O bond. Additionally, a broad band around 1700 cm^−1^ is observed, which is attributed to the bending vibration of H–O–H bonds from adsorbed water molecules on the material’s surface. Indeed, the spectrum displays features typical of amorphous niobium oxide, notably the bands centered at approximately 600 and 900 cm^−1^, along with vibrational modes associated with surface hydroxyl groups and adsorbed water in the regions around 1650 cm^−1^ and 3500–3000 cm^−1^, respectively [32]. A band at 3040 cm^−1^ is related to Nb-OH vibrations [16,18]. Interestingly, the absence of bands near 2900 cm^−1^, which are usually assigned to C–H stretching vibrations in alkyl groups, suggests complete decomposition of oxalate precursors during the solvothermal synthesis process. Finally, the presence of a vibration band at 1400 cm^−1^ in the non-calcined samples could be related to possible carbonate species adsorbed on the surface [33].

Upon treatment with H_2_O_2_, a marked reduction in the intensity of the Nb–O vibrational bands is observed, indicating partial removal or transformation of the surface Nb_2_O_5_ phase. Although weak vibrational features attributed to superoxo and peroxo species—typically appearing around 1140 and 890 cm^−1^, respectively—have been previously reported [34], only an increase in the band located at 880 cm^−1^ is detected, which can be related to the formation of peroxo species. On the other hand, the bands at 1700 cm^−1^ and 1400 cm^−1^, associated with H–O–H and carbonate species, are slightly reduced upon H_2_O_2_ treatment. This may suggest the removal of carbonate species from the surface, as well as the conversion of some –OH at Brønsted sites into peroxo species as described in [35]. However, the overall intensity of adsorbed water bands in the 3500–3000 cm^−1^ region increases, along with an increment of the Nb-OH band at 3040 cm^−1^. This, considering the reduction in the Nb=O band at 715 cm^−1^, indicates that the peroxo groups are mainly formed from the conversion of Nb=O bonds, as indicated in [20].

Moreover, after calcination at 900 °C, all bands corresponding to hydroxyl groups are absent, consistent with the expected dehydration and consolidation of the oxide network at high temperatures. The obtained bands are consistent with those obtained for previously reported Nb_2_O_5_ samples, prepared through microwave-assisted hydrothermal synthesis method and further treated at 800 °C [16]. Finally, after calcination at 1100 °C, the expected stabilization of the H-Nb_2_O_5_ polymorph [36] is indeed observed. Thus, the bands observed in the 800–1000 cm^−1^ and 400–800 cm^−1^ ranges can be related to the terminal stretching mode of Nb=O and to the symmetrical Nb_2_O_5_ stretching, respectively. These assignments are in good agreement with previously reported results for the monoclinic high-temperature H-Nb_2_O_5_ polymorph [37] and the measured Raman spectrum.

**Figure 5:**
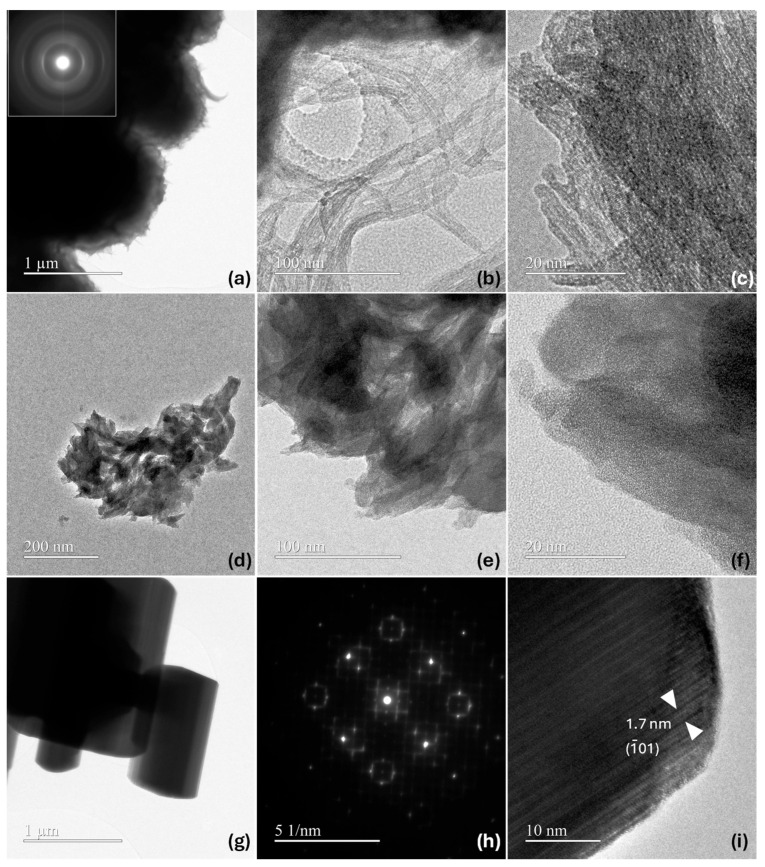
Representative TEM results: (**a**–**c**) TEM images at different magnifications for Nb_2_O_5_-as-prepared (corresponding ED pattern as inset in a); (**d**–**f**) TEM images for Nb_2_O_5_-H_2_O_2_; (**g**) TEM image for Nb_2_O_5_-1100 and (**h**) corresponding ED, (**i**) HRTEM image for Nb_2_O_5_-1100.

Figure 5 shows representative TEM images for the prepared samples: Nb_2_O_5_-as-prepared, Nb_2_O_5_-H_2_O_2_, and Nb_2_O_5_-1100. In the case of the Nb_2_O_5_-as-prepared sample (Figure 5a–c), micro-coils composed of interconnected fibers approximately 5 nm in diameter and exceeding 60 nm in length are observed. The corresponding electron diffraction (ED) pattern (inset in Figure 5a) is consistent with a nanoparticulated material with low crystallinity. In high-magnification HRTEM images, the interconnectivity and morphology of such fibers can be appreciated. These observations are consistent with the layered-like structure expected from XRD measurements. In contrast, this fibrous aspect of the particles becomes less defined in the Nb_2_O_5_-H_2_O_2_ sample (Figure 5d–f), resulting in a more amorphous appearance of the material. As deduced from XRD analysis, the treatment with H_2_O_2_ induces modification in the interlayer planes of the niobic acid and therefore in the material crystallinity. Indeed, this modification is clearly observed by comparing HRTEM images of Nb_2_O_5_-as-prepared and Nb_2_O_5_-H_2_O_2_ samples (Figure 5c,f). In this sense, the interaction of the material with hydrogen peroxide leads to a clear modification of its morphology. In this sense, the interaction of the material with hydrogen peroxide leads to a clear modification of the material morphology. EDX measurements performed on both samples confirm the presence of Nb and O (in both the spheres and the connecting fibers), with a negligible carbon signal. Figure S3 shows representative spectra. A markedly different situation is observed when the sample is calcined at 1100 °C (Figure 5g–i). In this case, the particles aggregate and grow, leading to the formation of micrometer-sized rod-like structures with high crystallinity. High-magnification images and electron diffraction patterns are consistent with those observed in the previously reported defective H-Nb_2_O_5_ phase [38]. Thus, the ED pattern (Figure 5h) is coherent with the short-range ordered region of crystal in Figure 5g along [010] direction and contrasts in the HRTEM image in Figure 5i are concordant with the expected interplanar distances for the H-Nb_2_O_5_, corresponding to standard PDF No. 037–1468 file.

**Figure 6:**
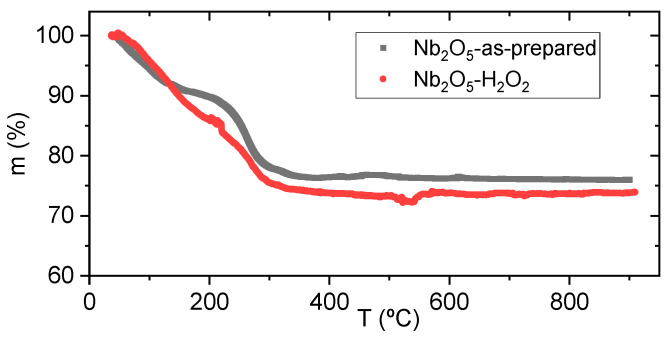
TG curves, for Nb_2_O_5_-as-prepared and Nb_2_O_5_-H_2_O_2_ samples.

Figure 6 presents the thermogravimetric (TG) curves for both Nb_2_O_5_-as-prepared and Nb_2_O_5_-H_2_O_2_ samples. In each case, a weight loss of approximately 74% is observed between room temperature and 300 °C. This mass loss is attributed to the release of physically adsorbed and structurally bound water, corresponding to the transformation of Nb_2_O_5_·nH_2_O into Nb_2_O_5_. Notably, the main difference between the as-prepared and H_2_O_2_-treated samples is the appearance of a shoulder around 200 °C. This feature may be associated with multiple desorption steps involving various surface species. Beyond this range, from 300 to 900 °C, the weight remains virtually unchanged, within the limits of experimental error, consistent with previously reported findings [39].

**Figure 7:**
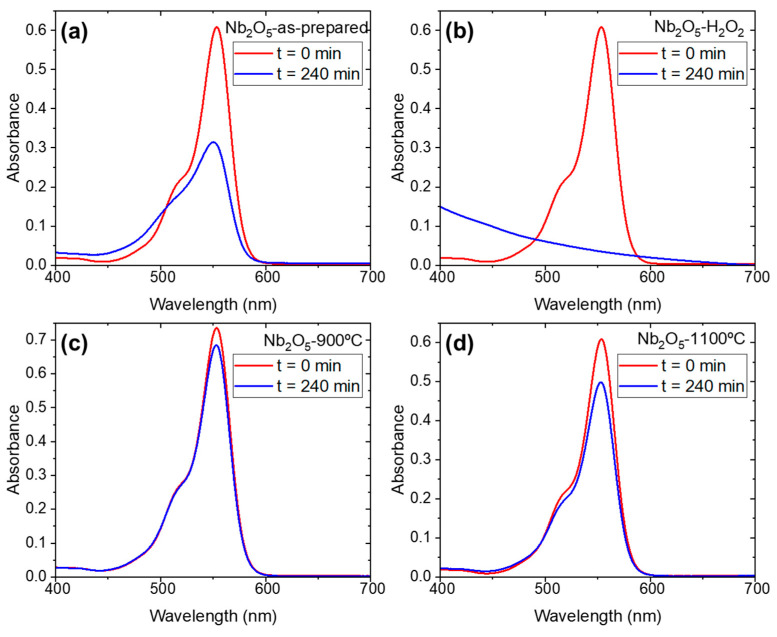
Photocatalysis degradation of Rhodamine B after 4 h of irradiation of UV light (365 nm and 405 nm): (**a**) Nb_2_O_5_-as-prepared; (**b**) Nb_2_O_5_-H_2_O_2_; (**c**) Nb_2_O_5_-900; (**d**) Nb_2_O_5_-1100.

Once the basic characterization of the samples was performed, the photocatalysis performance of the different samples was tested under UV irradiation. The results of the variation of RhB absorption after 4 h of irradiation are gathered in Figure 7. It is clearly seen that the low-temperature samples (as-prepared and H_2_O_2_, Figure 7a,b) show higher degradation efficiency than the high-temperature samples (900 °C and 1110 °C, Figure 7c,d). In the low-temperature samples, the structural defects and functional groups located at the surface are more efficient for catalysis than in more ordered, free-of-functional groups, high-temperature phases. This is, for the Nb_2_O_5_-H_2_O_2_ sample, RhB degradation is completed, whereas the degradation efficiency for Nb_2_O_5_-900 and Nb_2_O_5_-1100 does not reach 20% after 4 h. This observation agrees with previous studies when comparing TT and T-phases [13,15]. The transformation from TT- to T- and eventually H-Nb_2_O_5_ phases is commonly associated with a reduction in surface area, primarily due to crystallite growth and structural densification during calcination. This trend was reported in previous studies [14], in relation to the Raman spectra, and aligns with our observations. In our case, the decrease in photocatalytic activity observed for the high-temperature phases is consistent with a loss of surface-active sites, as supported by FTIR and Raman spectroscopy. These findings suggest that thermal treatment not only alters the crystallographic structure but also significantly impacts the surface reactivity of the material (by losing the active species at the surface, e.g., hydroxyl and peroxo groups).

Moreover, the four samples were tested under visible irradiation for 4 h. The results again confirm that no degradation is observed for the high-temperature phases, whereas low-temperature samples can degrade RhB under this illumination. Then, the reaction kinetics were studied for Nb_2_O_5_-as-prepared and Nb_2_O_5_-H_2_O_2_ samples.

**Figure 8:**
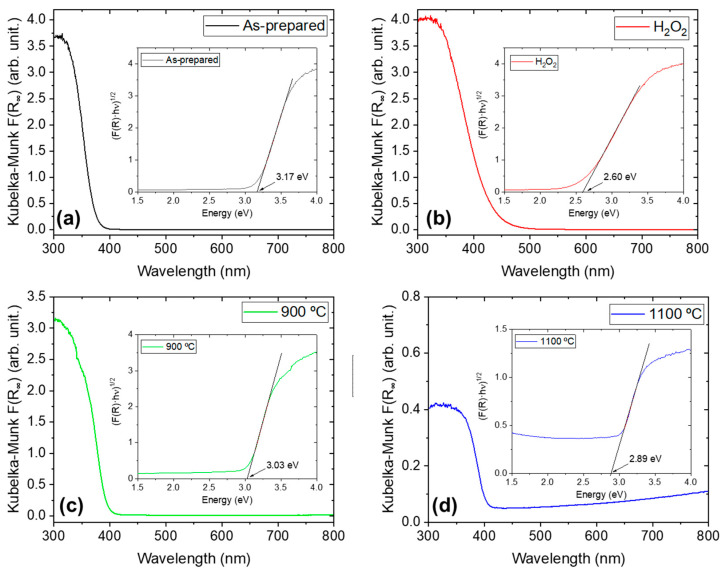
DRS measurements for (**a**) Nb_2_O_5_-as-prepared; (**b**) Nb_2_O_5_-H_2_O_2_; (**c**) Nb_2_O_5_-900; (**d**) Nb_2_O_5_-1100.

Since both low-temperature samples exhibit efficient photocatalytic degradation, DRS measurements were performed to determine the optical range in which the powders absorb light. The results are shown in Figure 8. The as-prepared sample displays an absorption edge well into the UV region (Figure 8a), whereas the Nb_2_O_5_-H_2_O_2_ sample shows a shift toward the visible region (Figure 8b). This observation is consistent with the yellowish color of the latter and aligns with the behavior reported in other studies involving peroxo species on the surface [40]. Upon thermal treatment, the absorption edge shifts towards a lower wavelength (Figure 8c,d), a behavior observed in other Nb_2_O_5_ materials [16]. The absorption edges determined from the Kubelka–Munk function and the Tauc plots [41] are 3.17 eV (391 nm) for the as-prepared sample, 2.60 eV (477 nm) for the H_2_O_2_ treated sample, 3.03 eV (409 nm) and 2.89 eV (429 nm) for the samples treated at 900 and 1100 °C, respectively.

**Figure 9:**
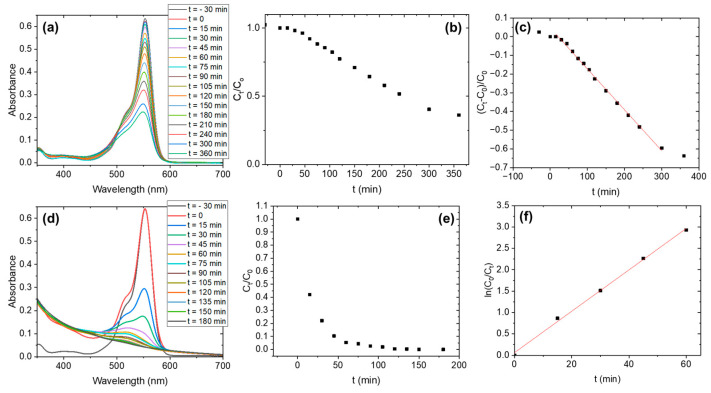
Photocatalysis kinetics under UV illumination: (**a**–**c**) Nb_2_O_5_-as-prepared; (**d**–**f**) Nb_2_O_5_-H_2_O_2_.

**Figure 10:**
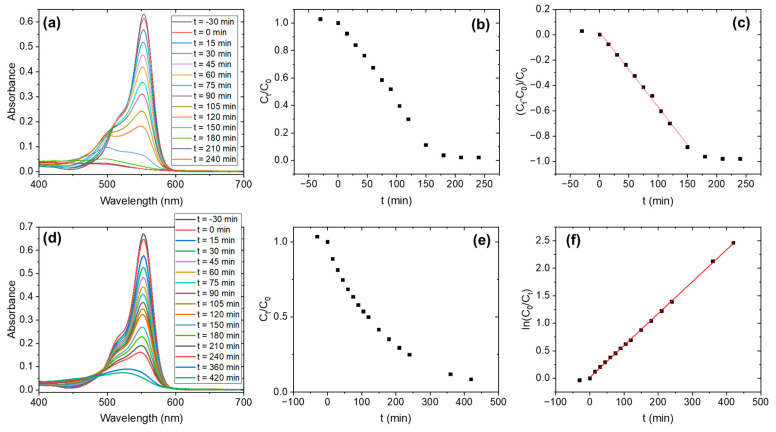
Photocatalysis kinetics under visible illumination: (**a**–**c**) Nb_2_O_5_-as-prepared; (**d**–**f**) Nb_2_O_5_-H_2_O_2_.

The evolution of the absorbance of the RhB solution in contact with both samples is presented in Figure 9 (under UV irradiation) and Figure 10 (under visible irradiation). In each case, the absorbance spectra over time are shown (Figure 9a,d and Figure 10a,d), along with the relative concentration at time *t* (C_t/_C_0_) (Figure 9b,e and Figure 10b,e), as well as the fit of the data according to the best pseudo-order kinetic identified (Figure 9c,f and Figure 10c,f). and the kinetic data fitted to the best-fitting pseudo-order model (Figure 9c,f and Figure 10c,f). Relative concentration is directly related to the absorbance ratio, in accordance with Beer’s law.

Considering that the amount of all the reactants involved, except for RhB, is unlimited, the catalyzed reaction will follow a rate law as follows [42,43]:

(2) $$v=\frac{dC_{t}}{dt}=-k_{a}C_{t}^{n}$$

where v is the reaction rate, $C_{t}$ is the concentration of RhB at time *t*, *n* is the pseudo reaction order and $k_{a}$ is the apparent rate coefficient. It is generally considered that other parameters that can influence the reaction (such as light intensity, temperature, etc.) are kept constant during the reaction, and their effect is included in the $k_{a}$ value. Then, the integration of Equation (2) leads to (for n≠1):

(3) $$C_{t}^{1-n}-C_{0}^{1-n}=\left(n-1\right)k_{a}t$$

In the case of pseudo-zero-order degradation reaction:

(4) $$n=0\;⟹C_{t}-C_{0}=-k_{a}t$$

(5) $$\frac{C_{t}-C_{0}}{C_{0}}=-\frac{k_{a}}{C_{0}}t$$

And for a pseudo-first-order degradation reaction:

(6) $$n=1\;⟹\;C_{t}=C_{0}e^{-k_{a}t}\;$$

(7) $$ln\left(\frac{C_{0}}{C_{t}}\right)=k_{a}t$$

The different orders of the degradation reaction are related to the strength of the rate of adsorption as a limiting factor for the reaction evolution. When the rate of degradation is well above the rate of adsorption, photocatalysis follows a pseudo-first-order reaction [43]. This is the behavior typically employed in photocatalysis studies. On the other hand, when the degradation reaction is limited by the rate of adsorption, the order of the reaction can vary from zero to two. In these cases, adsorption models, such as Langmuir–Hinshelwood model, must be considered in substitution of the $k_{a}$ [43].

The pseudo-order of the degradation reaction is different for the as-prepared sample and the H_2_O_2_ sample. By comparing the (C_t/_C_0_) vs. t curves, it is determined that the as-prepared sample follows a pseudo-zero order, whereas the H_2_O_2_ sample follows a pseudo-first-order. This tendency is observed for UV illumination as well as for visible illumination. Then, Equation (5) is used to model the behavior of the as-prepared sample (Figure 9c and Figure 10c) and Equation (7) is employed for the H_2_O_2_ sample (Figure 9f and Figure 10f). From the linear fittings, the reaction coefficients can be determined, and from them, the half-life time $t_{1/2}$ can be calculated with the equations:

(8) $$t_{1/2}=\frac{2^{n-1}-1}{\left(n-1\right)k_{a}}\cdot C_{0}^{1-n}$$

The results are summarized in Table 2. This table also includes the time at which we observed the complete disappearance of RhB (t_final_) as indicated by the absorbance spectrum (in coincidence with the total discoloration of the solution).

We observed that the H_2_O_2_ sample follows a pseudo-first-order degradation reaction, the most commonly observed for photocatalytic reaction. The t_1/2_ reaches a value as short as 14.3 min when the sample is irradiated with the UV lamps (365 + 405 nm) and decreases to 119.5 min when it is illuminated with the visible lamps (wavelength range between 420–750 nm). This decrease can indicate that both UV and visible under 477 nm (measured absorption edge) are activating the catalyst. Then, the degradation rate decreases for visible illumination, as not all the wavelengths in that region are activating the catalyst. In the case of the samples treated at 900 °C and 1100 °C, their absorption edges suggest they should be activated under UV illumination. However, although some degradation is observed under UV light, their photocatalytic activity remains low compared to the low-temperature samples. This indicates that the surface chemistry of Nb_2_O_5_ plays a more critical role than light absorption alone in determining the material’s photocatalytic performance. The shift of the absorption edge toward longer wavelengths in the Nb_2_O_5_-1100 sample could explain the slight difference in performance between the two high-temperature samples.

The case of the as-prepared sample is more intriguing. As pointed out by the diffuse reflectance measurements, the absorption edge of this sample is around 391 nm. Then, it should be more effective under UV light than under visible illumination, but we observe the opposite behavior. Additionally, the pseudo-order of the degradation reaction is zero, which can provide some clues about the behavior of this sample. Pseudo-zero-order kinetics is generally related to a saturation of the catalyst surface by reactants, i.e., there is another limiting factor apart from the rate of degradation [44]. In both samples, different functional groups at the surface of the photocatalyst may be behind this behavior. We discarded a photolysis process (which can be also a pseudo-zero-order process) by checking the negligible degradation of RhB under both illuminations in 4 h, and the experimental conditions are the same for all the tests.

On the other hand, since we are using a catalyst load of 2.5 ppm and an RhB concentration of 0.4 g/L (values comparable to those used in other studies) together with the behavior observed for the H_2_O_2_ sample, we can rule out the possibility that the pollutant concentration exceeds the catalyst’s capacity. Then, the observed behavior appears to be more closely related to the limited hydroxyl groups available in the as-prepared sample.

In the non-calcined samples, hydroxyl groups are present on the surface, with a higher concentration observed in the H_2_O_2_-treated sample. This increase is attributed to the interaction with aqueous H_2_O_2_, which promotes the formation of ROS (hydroxyl and peroxo groups) on the Nb_2_O_5_ surface, as confirmed by FTIR and Raman analyses [20]. This interaction induces partial amorphization of surface niobium octahedra, as evidenced by Raman and HRTEM measurements. These structural modifications contribute to an increased density of active sites. As previously reported, the photocatalytic performance of niobium-based materials correlates strongly with their ability to generate surface superoxo species, which are crucial intermediates in the formation of other highly reactive species such as singlet oxygen [21]. Therefore, the enhanced photocatalytic activity observed for the Nb_2_O_5_–H_2_O_2_ sample can be attributed to the presence of surface peroxo groups, as confirmed by Raman spectroscopy (Figure 3b). Additionally, a higher amount of water molecules intercalated within the micro-coil structure, as indicated by FTIR and HRTEM measurements, may further enhance the number of available active sites.

Regarding the influence of the illumination wavelength, it appears to be related to the redox potentials of the peroxo and hydroxyl species. The redox potential of peroxo species lies near the bottom of the conduction band of the semiconductor, whereas the hydroxyl redox potential is close to the top of the valence band [45,46]. Since Nb_2_O_5_ has a band gap in the UV region, UV light is required to have electrons to exchange with peroxo species. Conversely, visible light facilitates the transfer of electrons from adsorbed pollutant molecules on the hydroxyl species [33].

Considering all the previous observations and the previous literature, the proposed mechanism for RhB degradation is:

(9) $${Nb}_{2}O_{5}+h\nu ⟶Nb_{2}O_{5}+e^{-}+h^{+}$$

(10) $$h^{+}+OH^{-}⟶OH^{\cdot }$$

(11) $$RhB+OH^{\cdot }⟶intermediate\;compounds$$

In the case of the H_2_O_2_ treated sample, peroxo groups oxidate RhB:

(12) $$RhB+O_{2}^{2-}⟶intermediate\;compounds$$

Peroxo species can be recovered by catching electrons produced in the first step (Equation (9)).

Finally, in Table 3, we present a comparison of our results with previous works performed on Nb_2_O_5_ degradation of RhB, showing the good performance of the prepared nanomaterials in this study. Note the importance of considering the experiment parameters in the comparison.

## 4. Conclusions

In this work, we synthesized Nb_2_O_5_ nanomaterials using a solvothermal method, with niobium oxalate hydrate (V) as the precursor. The resulting material consists of micro-coils composed of interconnected nanometer-scale fibers. The as-prepared samples primarily crystallized as a mixture of the TT-Nb_2_O_5_ phase and niobic acid, with hydroxyl groups and adsorbed water present on the surface. Upon treatment with H_2_O_2_, peroxo species (O_2_^2−^) formed on the material’s surface. Subsequent thermal treatments at 900 °C or 1100 °C resulted in the removal of surface species and densification of the material. The differences in crystal structure and surface chemistry among the samples led to variations in photocatalytic performance. The H_2_O_2_-treated nanoparticles exhibited the highest photocatalytic activity, while the calcined samples were practically inactive. This loss of activity is attributed to the elimination of surface-active sites during thermal treatment and phase transformation. The differences in photocatalytic performance between the as-prepared and H_2_O_2_-treated samples under different illumination wavelengths are associated with the nature of the active surface species: peroxo species in Nb_2_O_5_-H_2_O_2_ samples are more effective under UV illumination, while hydroxyl species in the Nb_2_O_5_-as-prepared samples perform better under visible light.

## Figures and Tables

**Table 1:** Notation and synthesis characteristics of the prepared samples.

Sample Name	Preparation
Nb_2_O_5_-as-prepared	As prepared white powders from solvothermal synthesis
Nb_2_O_5_-H_2_O_2_	As prepared powders treated with H_2_O_2_
Nb_2_O_5_-900	As prepared powders thermal treated at 900 °C
Nb_2_O_5_-1100	As prepared powders thermal treated at 1100 °C

**Table 2:** Results obtained from the fitting of the photocatalysis data to the different kinetic models.

Sample	Irradiation	Order	$k_{a}$ (min^−1^)	t_1/2_ (min)	t_final_ (min)
Nb_2_O_5_-as-prepared	UV	0	0.00219·C_0_	228.3	-
Nb_2_O_5_-H_2_O_2_	UV	1	0.04838	14.3	~75
Nb_2_O_5_-as-prepared	Vis	0	0.00591·C_0_	84.6	~180
Nb_2_O_5_-H_2_O_2_	Vis	1	0.0058	119.5	-

**Table 3:** Comparison Nb_2_O_5_ performance for RhB degradation.

Material	Illumination	Catalyst Dosage	RhB	Performance	Reference
Nb_2_O_5_ micro-coils of nanofibers in H_2_O_2_	365 nm + 405 nm 25 W × 2	0.4 g/L	2.5 ppm	100%, 75 min	This work
Nb_2_O_5_ micro-coils of nanofibers	420–750 nm 35 W × 2	0.4 g/L	2.5 ppm	100%, 180 min	This work
Nb_2_O_5_ nanoparticles	254 nm (UVC) 15 W × 6	1 g/L	1 × 10^5^ M	99%, 60 min	[47]
Nb_2_O_5_ particles (T + low crystallinity)	254 nm (UVC) 15 W × 5	1 g/L	1 × 10^5^ M	100%, 60 min	[48]
Nb_2_O_5_ particles (low crystallinity)	254 nm (UVC) 15 W × 5	1 g/L	1 × 10^5^ M	100%, 90 min	[13]
T-Nb_2_O_5_ nanoparticles	UV Hg lamp 400 W	0.5 g/L +H_2_O_2_	10 mg/L	99%, 2 min	[49]
Nb_2_O_5_ nanorods	365 nm 8 W	1 g/L +H_2_O_2_	15 mg/L	92.5%, 30 min	[21]

## Data Availability

The original contributions presented in the study are included in the article, further inquiries can be directed to the corresponding authors.

## Supplementary Materials

The following supporting information can be downloaded at: https://www.mdpi.com/article/10.3390/nano15110846/s1. Figure S1. Emission spectra of the LED strips employed in photocatalysis experiments: (a) UV; (b) visible strips. Figure S2. Raman spectrum of the niobium oxalate hydrate (V) precursor. Figure S3. Representative EDX spectra for Nb2O5-as-prepared and Nb2O5-H2O2 samples. Cu signal is related to the grid.
