# Supplementary material for: Phase-Dependent Photocatalytic Activity of Nb2O5 Nanomaterials for Rhodamine B Degradation: The Role of Surface Chemistry and Crystal Structure
Source: Nanomaterials (Basel). 2025 Jun 1;15(11):846. doi: 10.3390/nano15110846 (PMC12157932; doi:10.3390/nano15110846)
Supplement: Supplementary file 1 [file nanomaterials-15-00846-s001.zip › nanomaterials-3647722-supplementary.pdf]

nanomaterials

Supplementary Material

Phase-Dependent Photocatalytic Activity of Nb_2O_5 Nanoparticles: The Role of Surface Chemistry and Crystal Structure

Aarón Calvo-Villoslada[1], Inmaculada Álvarez-Serrano[2], María Luisa López[2], Paloma Fernández[1], Belén Sotillo[1,*]

[1] Department of Material Physics, Faculty of Physics, Complutense University of Madrid, 28040, Madrid, Spain
[2] Department of Inorganic Chemistry, Faculty of Chemistry, Complutense University of Madrid, 28040, Madrid, Spain
* Correspondence: bsotillo@ucm.es

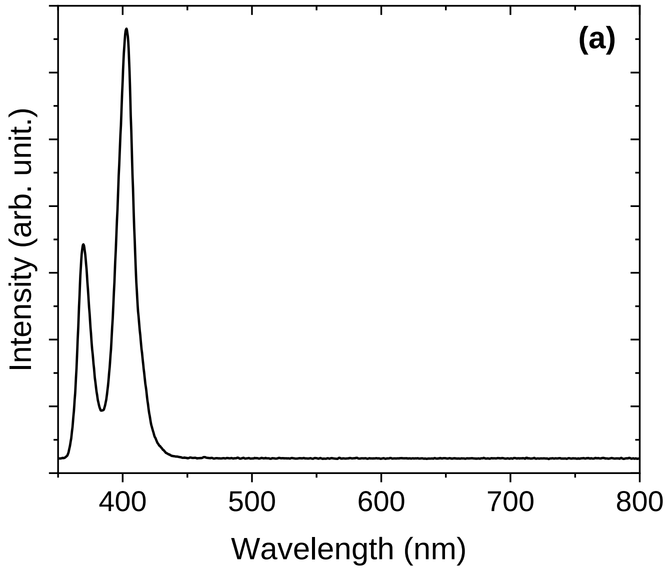

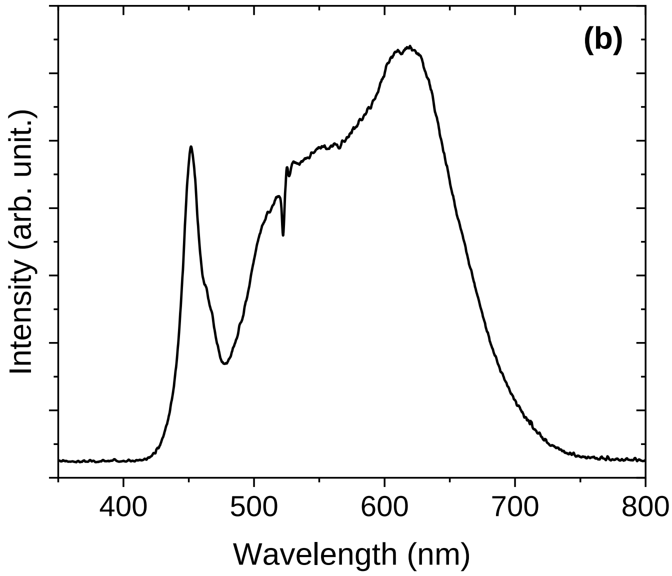

Figure S1. Emission spectra of the LED strips employed in photocatalysis experiments: (a) UV; (b) visible strips.

 https://doi.org/10.3390/nano15110846

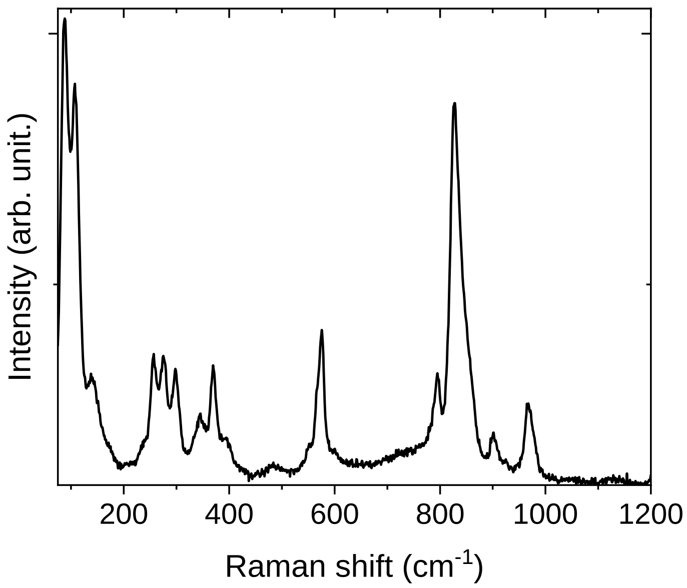

Figure S2. Raman spectrum of the niobium oxalate hydrate (V) precursor.

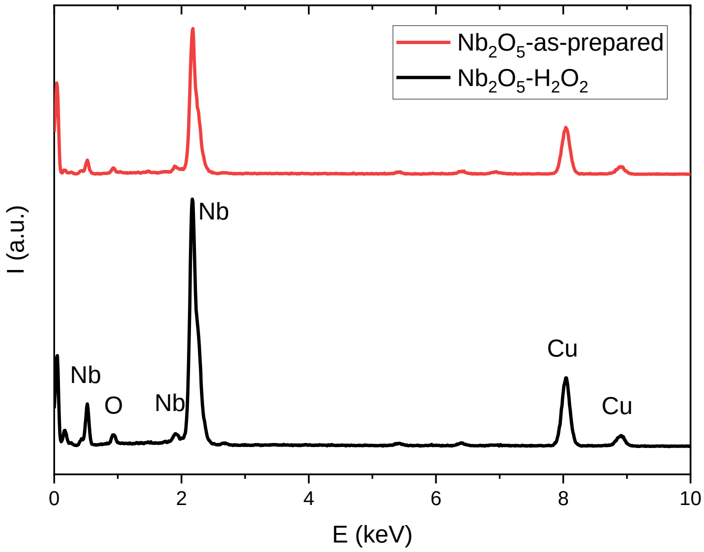

Figure S3. Representative EDX spectra for Nb_2O_5-as-prepared and Nb_2O_5-H_2O_2 samples. Cu signal is related to the grid.
